# Energetic basis for bird ontogeny and egg-laying applied to the bobwhite quail

**DOI:** 10.1093/conphys/coac063

**Published:** 2022-09-20

**Authors:** Nina Marn, Konstadia Lika, Starrlight Augustine, Benoit Goussen, Markus Ebeling, David Heckmann, Andre Gergs

**Affiliations:** Division for Marine and Environmental Research, Rudjer Boskovic Institute, 10002 Zagreb, Croatia; School of Biological Sciences, The University of Western Australia, Crawley WA 6009, Australia; Department of Biology, University of Crete, 70013 Heraklion, Greece; Akvaplan-niva, Fram High North Research Centre for Climate and the Environment, 9296 Tromsø, Norway; ibacon GmbH, 64380 Roßdorf, Germany; Bayer AG Crop Science Division, 40789 Monheim am Rhein, Germany; Bayer AG Crop Science Division, 40789 Monheim am Rhein, Germany; Bayer AG Crop Science Division, 40789 Monheim am Rhein, Germany

**Keywords:** Birds, Colinus virginianus, Dynamic Energy Budgets, metabolism, reproduction, sustainable management

## Abstract

Birds build up their reproductive system and undergo major tissue remodeling for each reproductive season. Energetic specifics of this process are still not completely clear, despite the increasing interest. We focused on the bobwhite quail — one of the most intensely studied species due to commercial and conservation interest — to elucidate the energy fluxes associated with reproduction, including the fate of the extra assimilates ingested prior to and during reproduction. We used the standard Dynamic Energy Budget model, which is a mechanistic process-based model capable of fully specifying and predicting the life cycle of the bobwhite quail: its growth, maturation and reproduction. We expanded the standard model with an explicit egg-laying module and formulated and tested two hypotheses for energy allocation of extra assimilates associated with reproduction: Hypothesis 1, that the energy and nutrients are used directly for egg production; and Hypothesis 2, that the energy is mostly spent fueling the increased metabolic costs incurred by building up and maintaining the reproductive system and, subsequently, by egg-laying itself. Our results suggest that Hypothesis 2 is the more likely energy pathway. Model predictions capture well the whole ontogeny of a generalized northern bobwhite quail and are able to reproduce most of the data variability via variability in (i) egg size, (ii) egg-laying rate and (iii) inter-individual physiological variability modeled via the zoom factor, i.e. assimilation potential. Reliable models with a capacity to predict physiological responses of individuals are relevant not only for experimental setups studying effects of various natural and anthropogenic pressures on the quail as a bird model organism, but also for wild quail management and conservation. The model is, with minor modifications, applicable to other species of interest, making it a most valuable tool in the emerging field of conservation physiology.

## Layman summary

An energy budget bird model with an egg-laying module is used to elucidate the energy fluxes and the fate of additional ingested energy associated with reproduction. Predictions capture well the ontogeny and egg-laying of the bobwhite quail and suggest that extra assimilates are associated with increased metabolic costs.

## 1 Introduction

Birds (class Aves or Neornithes) evolved from reptiles roughly 150 million years ago, and with roughly 10 000 species are the most diverse of all terrestrial vertebrates (Raven & Johnson, 2001). Despite the huge diversity, all birds — like their reptilian ancestors — have internal fertilization and lay eggs. Building up of the reproductive system and major tissue remodeling for each breeding season, is, however, specific to birds (Vezina & Salvante, 2010).

In the period directly preceding the egg incubation and the chick brooding period, reproductive organs of both males and females undergo recrudescence. During this period of rapid growth, the atrophied gonads increase in size up to several orders of magnitude (Vezina & Salvante, 2010). In passerine birds, the (mass-corrected) resting metabolic rate (RMR) of egg-laying females increases by 16%–24% compared to that of non-breeding females (Table 3 in Vezina & Salvante, 2010); for the quail the relative increase in RMR could be close to 60% (Pick *et al.*, 2016). All together, this implies major changes in energy demands and energy allocation, but energetic specifics of the gonad recrudescence and egg-laying are still not completely clear.

Recent review on passerine birds suggests that birds can offset some part of the increased energetic demands by changing their behaviour, as food supplies in seasonal environments are, during this period, often still relatively low (Vezina & Salvante, 2010). Commercial birds like chickens, ducks, and quails often experience a more constant environment, and could physiologically differ from passerine birds. Therefore, they could cope differently with the increased energetic demands. This group, however, received thus far surprisingly little attention in attempts to study or quantify energetic costs of reproduction (Alisauskas & Ankney, 1994, Ward & Macleod, 1992), which is changing only recently (Pick *et al.*, 2016).

Bobwhite quail is a species of commercial and conservation interest, used also in ecotoxicological research (Brennan *et al.*, 2020, IUCN, B. I., 2016, Valverde-Garcia *et al.*, 2018). With new stressors emerging — both natural (climate change affecting global and local environmental conditions) and anthropogenic (new chemicals with potential effects on wildlife) — the need to understand a species’ physiology and therefore have the ability to anticipate a metabolic response is growing (Chown, 2012, Wood *et al.*, 2018) and conservation physiology is emerging as an ‘increasingly integrated and essential science’ (Cooke *et al.*, 2013).

Conservation in general, and conservation physiology in specific, need predictive models to fulfill their complex goals (Chown, 2012, Cooke *et al.*, 2013, Wood *et al.*, 2018). Good predictive models are based on underlying processes (growth but also maturation and reproduction) rather than empirical curve fitting. They are thus bypassing the frequent scarcity of data, while having the capacity to predict responses in an array of simulated conditions (Wood *et al.*, 2018). Frequently such mechanistic models seem complex and require a plethora of data to parameterize, resulting in potentially fewer researchers using the models (Jusup *et al.*, 2017, Wood *et al.*, 2018). Recently, however, more active attempts are being made to simplify the presentation of well-thought-out mechanistic models, while emphasizing their modular nature: the model should not be more complex than is necessary (Jusup *et al.*, 2017, Kearney, 2020). Dynamic Energy Budget (DEB) theory (Jusup *et al.*, 2017, Kooijman, 2010, Nisbet *et al.*, 2000, Sousa *et al.*, 2008, 2010) is an over-arching theory for general metabolic organization — from animals to unicellulars and plants — and as such is a base for several typified models (Marques *et al.*, 2018), the simplest one of which is the standard model applicable to the bobwhite quail (AmP, 2021).

The DEB-based Add-my-Pet collection on animal data and energetics (AmP, 2021) currently houses 503 bird species, including the bobwhite quail. All bird species are modeled by the standard (‘std’) DEB model, which captures well the general bird ontogeny and life cycle, but lacks specifics. This is because the standard DEB model aims for generality, and therefore does not *a priori* include detailed processes or activities specific for a species or a taxonomic group, such as details on the egg-laying process. Also, food is in the simple standard model assumed constant, whereas quail — like many other birds — change food types throughout ontogeny, and drastically increase food consumption prior to and during egg-laying (Brennan *et al.*, 2020, Cain & Lien, 1985). More nutritious food for juveniles generally results in more energy available for growth and maturation, speeding up the two processes. The fate of additional energy ingested by the adults as preparation for egg-laying, however, is less clear and is still open to debate. One hypothesis is that the energy and nutrients are used directly for egg production, as was observed in, e.g. barn swallows (Ward & Bryant, 2006). The other hypothesis is that the energy is mostly spent fueling the increased metabolic costs incurred by building up and maintaining the reproductive system, and then egg-laying itself (Pick *et al.*, 2016, Vezina & Salvante, 2010, Vezina & Williams, 2002). These processes (feeding, growth, maturation, and reproduction) and their interlinks, become especially important when the changing conditions might result in food shortages, or when the primary source of contaminants is in food, which may or may not have effects on the offspring. Because DEB models have explicitly formulated assumptions that follow rules for energy and mass allocation and conservation, they can readily be extended to include such specifics, while keeping the consistency.

Our aim has been to improve the predictive power and applicability of the standard DEB model for the birds in general, and bobwhite quail in specific, while taking into account the aforementioned aspects specific for quail and closely related species. Additionally, we wanted to explore potential sources of inter-individual variability. The data used to parameterize the model is collected on many different individuals, but the estimated parameter set of a model is meant to represent an average individual of a species. Therefore, running the model with the estimated parameter set does not reproduce the variability, but this *can* be done by incorporating variability into certain parameters. This, in turn, can elucidate the potential sources of the observed variability, which deepens the understanding of a species’ physiology and can be used for specific model applications.

We mainly focused on the reproductive part and early life stages of the bobwhite quail. In particular, we studied incubation period — characterized by incubation duration, embryo development and growth, and wet weight at hatching or birth — and growth of juveniles (hatchlings) during the first 2 weeks of age. These aspects of the early life stages are not only important end-points in standardized reproductive studies (OECD, 1984), but are also important aspects of wild quail life cycle (Brennan *et al.*, 2020, and references therein]. Reproduction period — characterized by increased food ingestion, egg production and changes in adult wet weight — has additional importance because it contains information about energy and nutrient allocation during the reproductive phase of birds in general.

In the following sections we first present the model organism (bobwhite quail), then follow to describe the main model assumptions and alterations from the standard DEB model for birds. To elucidate the energy flows during egg-laying, we formalized and tested the two hypotheses for allocation and use of additionally acquired energy (see Methods for details). Then we explore how the modified model, including the egg-laying module, predicts the ontogeny and reproduction of the reared bobwhite quail. Next, we select a more likely energy allocation hypothesis, and then use the model based on that hypothesis to explore potential sources of inter-individual variability observed in the data. Finally, the results are discussed in the context of possible applications and extensions.

### Northern bobwhite quail, *Colinus virginianus*

The northern bobwhite quail is one of the most intensively studied game birds in the world: it is economically important and valued for sport (hunting), for commercial egg and meat production, and for studying effects of micro-locations and habitat management on distribution and abundance of wild birds (Brennan *et al.*, 2020, and references therein]. Additionally, the northern bobwhite quail has been playing a major role in laboratory studies to test the physiological and behavioral effects of pesticides on wildlife (Quinn *et al.*, 2009, Valverde-Garcia *et al.*, 2018).

Conservation status according to the International Union for Conservation of Nature (IUCN) is Near Threatened, and some local populations are in decline due to, for example, agriculture or land mis-management (IUCN, B. I., 2016). Generally though, the species is quite resilient thanks to its relatively short life span — 1–5 years in the wild — and a decent reproductive output: first reproduction within a year from hatching, with up to 25 chicks in a nesting season (Brennan *et al.*, 2020). Captive-reared bobwhites can live 8–10 years, and even continue producing eggs during that period (Quinn *et al.*, 2009). Egg preparation and laying in the wild occurs in batches (up to two broods per nesting season), whereas in captivity eggs are usually taken from the hens daily to prolong egg production (Baldini *et al.*, 1952, Quinn *et al.*, 2009).

Size varies extensively across the species’ geographic range, with an average weight of around 180 g for adult bobwhites. Maximum recorded weight for wild quails is 255 g for males and 240 g for females, even though males are generally several grams lighter than females (Brennan *et al.*, 2020, and references therein]. ‘Adulthood’ for captive-reared quails is defined as attaining an adult body weight of at least 200 g (flight-type) or 400 g (meat-type) (Kato *et al.*, 2013), or being stimulated by light into producing at least one egg (Baldini *et al.*, 1952, Quinn *et al.*, 2009). Even though sexes are distinguishable at 8–10 weeks of age (Brennan *et al.*, 2020), it is generally considered that the bobwhite quails require 20–25 weeks to mature (Baldini *et al.*, 1952, Kato *et al.*, 2013, Quinn *et al.*, 2009).

Preparation for the reproductive season is induced by longer days: in the wild this happens in late spring, whereas in captivity it is regulated by artificial day–night cycles and therefore is independent of the time of the year (Baldini *et al.*, 1952, Quinn *et al.*, 2009, Schom & Abbott, 1974). During the 30–40 days period following the photostimulation, both males and females considerably increase their food intake, and the (female) reproductive system usually undergoes notable growth and enlargement resulting also in increased metabolic activity (Cain & Lien, 1985, Pick *et al.*, 2016).

## 2 Methods

### 2.1 Model setup and data

#### DEB model of the quail

To describe the ontogeny of the bobwhite quail (*C. virginianus*) we follow the same general assumptions for the standard DEB model as described in Kooijman (2010); model equations and parameters are summarized in Tables 1 and 2. Briefly, the state of the individual is defined by the state of (i) two main physical compartments: structure ($V$or $L$, with $V=L^3$) and reserve ($E$), and (ii) energy invested into maturity ($E_H$). The first two variables track the size of the organism, while the latter tracks it developmental stage (Augustine *et al.*, 2011, Kooijman, 2010). Standard DEB model has two metabolic switches: ‘birth’ is a maturity threshold signifying a metabolic switch from embryo (not reproducing and not feeding but maturing) into juvenile (feeding, maturing but not reproducing), and ‘puberty’ is a threshold for a switch from juveniles (maturing) into adults (reproducing). For birds, birth coincides with hatching, an additional maturity switch linked to fledging is introduced, and puberty in DEB context most often coincides with biological puberty but often precedes the onset of reproduction; age at maturity can therefore be independent of age at reproduction.

**Table 1:** State variables and dynamics of an individual. For parameters, see Table 2. Egg module equations are extensions of the standard DEB model, and differ between the two energy allocation hypotheses marked with H1 and H2 (see Fig. 1). Both of the hypotheses account for the up-regulated feeding prior to and during egg-laying, resulting in the additional assimilation flux $\dot {p}^{R}_A = s_X(t)\{\dot {p}_{Am} \}f L^2$, where $s_X$ is a monotonic increasing function with $0 \leq s_X \leq s_{max}$ (see text for details). Growth and mobilization are affected by the specific growth rate $\dot {r}$ (d$^{-1}$), defined below.

**State variable** (Units)	**Description**	**Dynamics**
$L$ (cm)	Structural body length	$\frac {d}{dt}L\,\, = \frac {[\dot {p}_G]}{3[E_G]} L = \dot {r}L/3$
$ E$ (J)	Reserve energy	$\frac {d}{dt}E\,\,\,$ $=\dot {p}_A-\dot {p}_C$
$ E_H$ (J)	Energy invested into maturation	$\frac {d}{dt}E_H$ $=\dot {p}_R(E_H<E_H^p)$
$ E_R$ (J)	Energy invested into reproduction	$\frac {d}{dt}E_R$ $= \dot {p}_R (E_H \ge E_H^p)$
**Process**	**Energy flux** (J.d$^{-1}$)	**Egg-module Eq.**
Assimilation:	$ \dot {p}_A= \{\dot {p}_{Am} \}f L^2(E_H \ge E_H^b) $	$\dot {p}_A= \dot {p}_A + \dot {p}^{R}_A$ (H2)
Mobilization:	$ \dot {p}_C =E(\dot {v}/L - \dot {r})$	
Somatic maintenance:	$ \dot {p}_S= {[\dot {p}_M]}L^3$	$ \dot {p}_S= {(1+s_X(t)f)[\dot {p}_M]}L^3$ (H2)
Maturity maintenance:	$ \dot {p}_J = \dot {k}_J E_H^p$	
Growth:	$ \dot {p}_{G} = \dot {r} [E_G] L^3$	
	$\dot {r}\textsuperscript {(a)} = \left \{ \begin {array}{ l } \frac {E \dot {v}/ L - [\dot {p}_M] L^3/ \kappa }{E + [E_G] L^3/ \kappa } \\[1ex] \quad \quad \footnotesize {\textrm {if } \kappa E \dot {v} \geq [\dot {p}_M] L^4}\\[1.5ex] \frac {E \dot {v}/ L - [\dot {p}_M] L^3/ \kappa }{E + \kappa _G [E_G] L^3/ \kappa } \\[1ex] \quad \quad \footnotesize {\textrm {otherwise}}. \end {array} \right .$	
Maturation/reproduction:	$ \dot {p}_R = (1-\kappa )\dot {p}_C-\dot {p}_J $	$ \dot {p}_R = (1-\kappa )\dot {p}_C-\dot {p}_J + \dot {p}^{R}_A$ (H1)

(a) The specific growth rate $\dot {r}$ (d$^{-1}$) is defined following starvation rules outlined in Section 4.1.5 of comments to Kooijman (2010). $\kappa _G \approx 0.8$ is the growth efficiency, representing here the inefficiency of degrading structure in order to cover the required maintenance costs (for other parameters, see Table 2).

**Table 2:** Standard DEB model primary and auxiliary parameters for the northern bobwhite quail, estimated for both energy allocation hypothesis (see Methods). Rate parameters are listed at the reference temperature 20 $^\circ $C unless otherwise noted. Notation: square brackets, [ ], indicate parameters normalized to structural volume, and curly brackets, { }, indicate parameters normalized to structural surface area.

Symbol	Units	Value (H1)	Value (H2)	Description
$z$	–	2.62	2.60	Zoom factor
$\left \{\dot {p}_{Am}\right \}$	J d$^{-1}$ cm$^{-2}$	1257	1339	Maximum specific assimilation rate
$\kappa _X$	–	0.47	0.46	Digestion efficiency (of food to reserve)
$\dot {v}$	cm d$^{-1}$	0.0190	0.0193	Energy conductance
$\kappa $	–	0.49	0.39	Allocation fraction to soma
$[\dot {p}_M]$	J d$^{-1}$ cm$^{-3}$	233.2	203.7	Somatic maintenance (volume specific)
$[E_G]$	J cm$^{-3}$	7320	7332	Specific cost for structure
$E_H^b$	J	1.077 $\cdot 10^4$	0.925 $\cdot 10^4$	Maturity at birth
$E_H^x$	J	$1.001 \cdot 10^6$	$ {1.258} \cdot 10^6$	Maturity at fledging
$E_H^p$	J	$1.002 \cdot 10^6$	1.279 $\cdot 10^6$	Maturity at puberty
$\delta _M$	–	0.101	0.109	Shape coefficient
$\delta _{Me}$	–	0.279	0.280	Shape coefficient for embryo data
$\delta _{Mt}$	–	0.5782	0.544	Shape coefficient for tarsus length
$f$	–	1	1	scaled functional response, $f$, for zero-variate data
$f_{NB}$	–	1.651	1.651	$f$ for Newsted *et al.* (2008) juvenile data
$f_{B}$	–	2.125	2.434	$f$ for Bayer (2008) juvenile data
$f_{JH}$	–	1.888	1.943	$f$ for Jones & Hughes (1978) juvenile data
$f_{L}$	–	2.134	2.134	$f$ for Lyon (1962) juvenile data
$E_{\mbox {\small {sperm}}}$	J	1.134$ \cdot 10^4$	1.667 $\cdot 10^4$	Energy in sperm, males
$\dot {k}_R$	d$^{-1}$	0.717	0.728	Rate of egg production at quail body temp. of 38.9$^{\circ }$C
$f_{mf}$	–	0.933	0.931	Scaled functional response for adults
$s_{max}$	–	1.434	0.987	Scaled functional response for up-regulation
				of feeding during reproduction

Other primary and auxiliary parameters with common values for both hypotheses: Arrhenius temperature, $T_A=9000$ K; maturity maintenance rate coefficient, $\dot {k}_J=0.002$ d$^{-1}$; reproduction efficiency, $\kappa _R=0.95 $; Weibull aging acceleration, $\dot {h}_a=1.82 \cdot 10^{-11}$ d$^{-2}$; Gompertz stress coefficient, $s_G=0.05$; specific density of dry structure, $d_{Vd} =0.28$ g/cm$^3$; molecular weight of dry reserve, $w_{Ed}=23.9$g/mol; chemical potential of reserve, $\mu _E=550$ KJ/mol.

Energy is assimilated from food into reserve, where food ingestion is assumed to follow a functional response relationship with food density and to be proportional to organism’s surface area. The scaled functional response for food, $f$, generally has a value between zero (no food) and 1 (abundant food), i.e. it is expressed relative to maximum food for a physiologically similar individual of the same size eating the same food. Assimilated energy is mobilized from reserve to fuel all other metabolic processes: a fixed part $\kappa $ of the mobilization flux $\dot {p}_C$ is allocated to the somatic branch, and the remaining $(1-\kappa )\dot {p}_C$ to the reproduction branch. The somatic branch fuels growth and maintenance of structure, whereas the reproduction branch fuels maturity maintenance and either increase of maturity (prior to puberty) or reproduction (post puberty). After puberty a third physical compartment, reproductive buffer ($E_R$), is introduced. Emptying of the reproduction buffer follows species-specific buffer handling rules (Kooijman & Lika, 2014); we expand on this in the ‘egg-laying module’ subsection.

DEB-defined compartments ($E$, $V$, $E_H$, $E_R$) are generalized compartments that do not directly correspond to specific body compartments, but are instead defined by their energy and mass dynamics (Kooijman, 2010). Structure ($V$) and maturity ($E_H$) generally only increase and require maintenance; a good example of structural or somatic maintenance would be protein turnover, while good examples for maturity maintenance would be maintenance of the general level of complexity and the immune system. The maintenance required to keep the whole organism functioning is fueled by mobilizing the reserve; should the mobilized energy be insufficient to cover maintenance costs, then starvation rules apply; we come back to this a bit later. Reserve ($E$), unlike structure and maturity, fluctuates in volume and available energy and does not require (an external energy source for) maintenance (Kooijman, 2010). For example, liver, often serving as an energy and material (lipid, protein) storage in birds, is interpreted as a mixture of structure and reserve. Reproductive buffer ($E_R$) is seen as a type of reserve in the sense that no energy is required for maintenance, and its amount fluctuates depending on the conditions (Kooijman, 2010). Ovaries and oviduct, for example, are a mixture of structure and reproductive buffer, where the structural part of the reproductive system requires maintenance, but all of the reproductive material does not. (For more details, please see Jusup *et al.*, 2014, Kooijman, 2010, Nisbet *et al.*, 2000, Sousa *et al.*, 2008, 2010.)

Body mass of an individual has contributions from structure ($V=L^3$), reserve ($E$), and (for reproducing adults) from reproduction buffer ($E_R$). Mass quantified as wet weight ($W_w$) is given by

(1) \begin{align*}& W_w = d_{Vw} L^3 + E \frac{w_{Ed} d_{Vw}}{\mu_E d_{Vd}}+ E_R \frac{w_{Ed} d_{Vw}}{\mu_E}, \end{align*}

where $ d_{Vw} $ and $ d_{Vd} $ are, respectively, the specific densities of wet and dry structure (g/cm$^3$); $ w_{Ed}$ the molecular weight of dry reserve (g/mol); and $\mu _E$ the chemical potential of reserve (J/mol) (Kooijman, 2010). It is assumed that the specific density of wet structure equals that of water (i.e. $ d_{Vw} =1~g/cm^3$) and that reproduction buffer does not include water to maximize energy density.

Physical (measured) length ($L_w$) is linked to the abstract structural length ($L$) by an auxiliary model parameter called the shape coefficient, which is estimated (by optimization routines) from data. The relationship is given by $L_w=L/\delta _M$; we use $\delta _M$ for total length, $\delta _{Mt}$ for tarsus length and $\delta _{Me}$ for embryo. Structural length is defined as the cubic root of structural volume: $L = V^{1/3}$.

When the total cost of maintenance is greater than the energy that can be mobilized from the reserve, then starvation occurs. In fully grown adults suddenly faced with increased energy demands linked with reproduction, starvation is in fact quite likely. We follow starvation rules outlined in Section 4.1.5 of comments to Kooijman (2010); see Table 1 for the equations. Growth rate during starvation is negative, which results in shrinking of structure. Starvation is generally not a part of the standard DEB model (Kooijman, 2010), but we include it to account for changes in food quality and availability experienced by the quails. The other non-standard change linked to food availability is assumed to affect juveniles, and later reproducing adults. We assume juveniles may experience different (than adults) food availability during the first 50–60 days of their life, to reflect the relatively fast growth rates observed in that period, as well as abundant food available to the chicks. We also assume that adults up-regulate their feeding prior to and during reproduction, as we present next.

#### Egg-laying module

To appropriately capture the physiological processes associated with reproduction, we developed an ‘egg-laying module’, i.e. an extension of the standard DEB model that explicitly tracks the energy assimilated during increased food ingestion prior and during egg-laying, and the energy deposited into eggs at a certain rate.

During a period $[t_0, t_1]$, starting before and including egg-laying, females (and males to an extent) increase feeding. The increase in feeding is modeled as up-regulation that results in the extra assimilation rate $\dot {p}^{R}_A$ proportional to a ‘stress’ factor $s_X$:

(2) \begin{align*}& \dot{p}^{R}_A = s_X(t)f\{\dot{p}_{Am} \} L^2. \end{align*}

The stress factor $s_X$ is a monotonic increasing function with $0 \leq s_X \leq s_{max}$, and for simplicity we assume a linear increase with time:

(3) \begin{align*} s_X(t) &= s_{max} \frac{t-t_0}{t_1-t_0} \textrm{~ for~ } t \in [t_0, t_1] \textrm{,~ and~ } s_X(t)\nonumber\\&=0 \textrm{~ for~ } t \not \in [t_0, t_1]. \end{align*}

**Figure 1:**
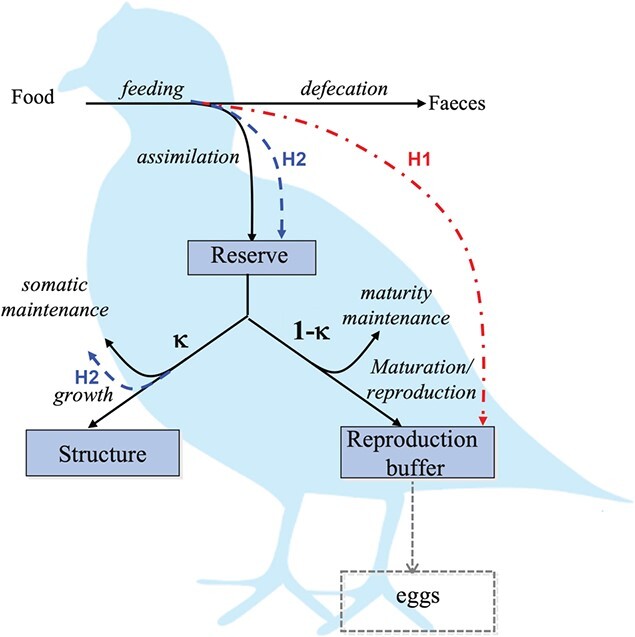
Conceptual representation of the metabolic processes. Solid arrows represent standard energy fluxes; dashed arrows represent up-regulated assimilation fluxes for the two energy allocation hypotheses; and boxes mark state variables. Energy is assimilated from food into reserves and subsequently allocated to fuel the metabolic processes: a fixed fraction $\kappa $ of the mobilized flux is allocated to somatic maintenance and growth, and the remaining ($1-\kappa $) to increase and maintain maturity or to reproduction. Two hypotheses for the energy pathways of the up-regulated feeding: the extra assimilates are directly allocated to reproduction buffer (Hypothesis 1 - H1, red dashed-dot line) or extra assimilates are added to the reserve and females increase feeding to compensate for an increase in somatic maintenance (Hypothesis 2 - H2, blue dashed lines).

The allocation of the extra assimilated energy depends on the energy pathway, for which two hypotheses were formulated as follows (see also Fig. 1 and Table 1):

**Hypothesis 1 (H1)**: The extra assimilates are directly allocated to egg production. This is modeled by directly adding them to the reproduction buffer, and the reproduction rate then becomes

\begin{align*}& \dot{p}_R = (1-\kappa)\dot{p}_C-\dot{p}_J + \dot{p}^{R}_A. \end{align*}

**Hypothesis 2 (H2)**: The extra assimilates are added to the reserve. Females increase feeding to compensate for an increase in somatic maintenance, and the increase in maintenance rate and feeding correlate with each other. The assimilation rate and the somatic maintenance rate become, respectively,

\begin{align*}& \begin{split} \dot{p}_A = (1+s_X(t))\{\dot{p}_{Am} \}f L^2 \textrm{and }\\ \dot{p}_S= {(1+s_X(t)f)[\dot{p}_M]}L^3. \end{split} \end{align*}

The energy assimilated from food is, in part either directly (H1) or indirectly (H2), allocated to reproduction. Adults first accumulate energy for reproduction in a buffer $E_R$ at a rate $\dot {p}_R$ (see Table 1); the emptying of $E_R$ is controlled by buffer handling rules. The process of egg-laying is a Poisson point process, i.e. events occur continuously and independently at a constant rate $\dot {k}_R$. The time between egg-laying, $t_e$, is assumed to be exponentially distributed with mean $\dot {k}_R ^{-1}$. Ovulation is triggered with photo-stimulation and happens at time intervals $t_e$ provided that $\kappa _R E_R \geq E_0$, where $E_0$ is egg size defined as initial energy in the egg, $\kappa _R$ is reproduction efficiency accounting for energy losses, or the cost of making an egg. If there is not enough energy when the time comes for ovulation, then the hen waits another $t_e$ period. Initial energy in an egg was calculated assuming maternal effect, i.e. that reserve density (defined as $[E]=E/L^3$) of the hatchling at birth is equal to that of the mother at egg-laying, $[E_b] = [E]$ (Kooijman, 2010, 1986). Variable feeding implies non-constant $[E]$ and, consequently, non-constant $[E_b]$. This also implies that a hen will lay larger eggs while experiencing a higher scaled functional response $f$. Therefore, $E_0$ calculated via the maternal effect will vary in time.

#### Data

To parameterize the model and then formulate and test assumptions for the egg-laying module, we combine general information available in the scientific literature, such as data points and datasets on life history traits, growth, and maturation, with more detailed data from scientific reports. The scientific reports are a valuable source of data for several reasons: (i) data are standardized — for example, control data of OECD 206 avian reproduction studies (OECD, 1984); (ii) data are collected on individuals often over several generations, with direct links between parents and offspring; (iii) temperature and food are regulated and reported, often in detail, removing a major source of uncertainty that is present in field data; and (iv) reared bobwhite quail used in laboratory studies and tests are still the same species as the wild bobwhite quail. Therefore, data from scientific reports can provide enough detail for hypothesis formulation and testing, but at the same time relate to the general quail physiology and can therefore be used to parameterize the DEB bobwhite quail model.

We use data from a reproduction toxicity study dealing with a compound that is a center point for several ongoing research modules (Bayer, 2008, report M-299245-02-1], but focus exclusively on the control data. The experiment was initiated when individuals were 112 days (16 weeks) old and they were kept at low light for 10 weeks, after which the length of day was increased (photo-stimulation). Photo-stimulation triggered the preparation for egg-laying and the first eggs were reported at the end of week 14 when individuals were roughly 210 days old. Egg-laying lasted until the experiment was terminated in experimental week 23, when birds were approximately 273 days old. The mean egg-laying rate was 5.03 eggs per week, resulting in a mean of 0.72 eggs per day or a mean of 1.39 days between full eggs. In addition to the egg-laying rate, the control data from the report provide weekly data on hatchling mass, 14-day-old chick mass, food ingestion per cage (i.e. bird pair) throughout the experiment, and adult mass before and after the egg-laying phase. The experiment (control) was replicated in 18 cages, with two adult birds (one male and one female) per cage, but one cage was excluded from the analysis because one of the birds died during the experiment.

#### DEB model calibration and hypotheses evaluation

The experimental setup of the study is ‘translated’ into the model as follows: individuals reach puberty sometime before the start of the experiment, presumably when they are roughly 100 days old (assumption based on Flores-Santin *et al.*, 2018). After this, we assume they immediately start investing energy into the reproduction buffer ($E_R$) at a rate $\dot {p}_R$ (Kooijman, 2010), but this does not yet result in laid eggs (birds did not start laying eggs in Flores-Santin *et al.*, 2018). Based on the data, we set the start of ingestion up-regulation as being 25 days before the start of egg-laying; during this period, we assume that the reproductive organs prepare (enlarge) (Cain & Lien, 1985, Vezina & Salvante, 2010), after which eggs are released. The egg-laying lasts while the quail has enough energy in the reproduction buffer for an egg. Depletion of the reproduction buffer occurs by egg release, and filling of the buffer occurs by investment of energy to reproduction (energy flux $\dot {p}_R$). The rate of depletion will depend on the size of the egg ($E_0$) and the interval between laying two eggs ($t_e$), both of which can vary. Egg-laying will presumably stop completely if a quail experiences a prolonged period during which the rate of energy depletion is larger than that of energy replenishment.

**Figure 2:**
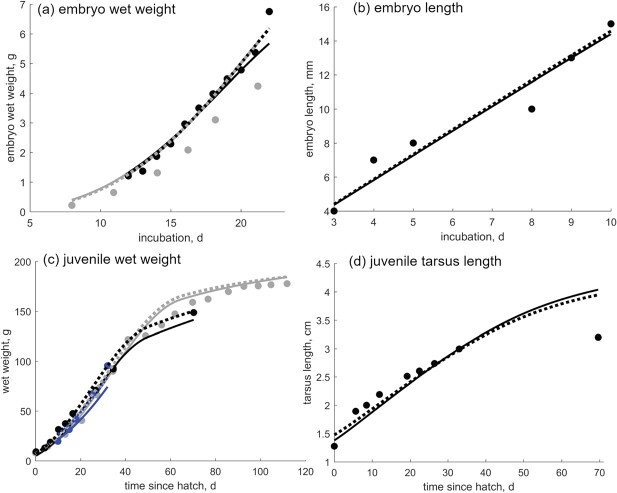
Data (dots) and model predictions (lines) for the following: (a) embryo wet weight, (b) embryo length, (c) juvenile wet weight over time, and (d) juvenile tarsus length over time. Model predictions obtained with Hypothesis 1 for energy allocation are plotted as dotted lines, and model predictions obtained with Hypothesis 2 are plotted as full lines. Dots of different colors mark different data sets. Data sources are as follows: embryo weight data, Schom *et al.* (1979) (black) and Flores-Santin *et al.* (2018) (gray); embryo length data, Hanson (1954); juveniles weight data, Lyon (1962) (black), Newsted *et al.* (2008) (blue) and Jones & Hughes (1978) (gray); and tarsus length data, Lyon (1962). Relevant parameters for model predictions are given in Table 2. A constant contribution of reserve to weight ($\omega $) is assumed for the whole life cycle, i.e. also to predict the embryo growth in wet weight (embryo without the yolk). A higher-than-reference scaled functional response $f$ is assumed for early juvenile growth (see Table 2).

**Figure 3:**
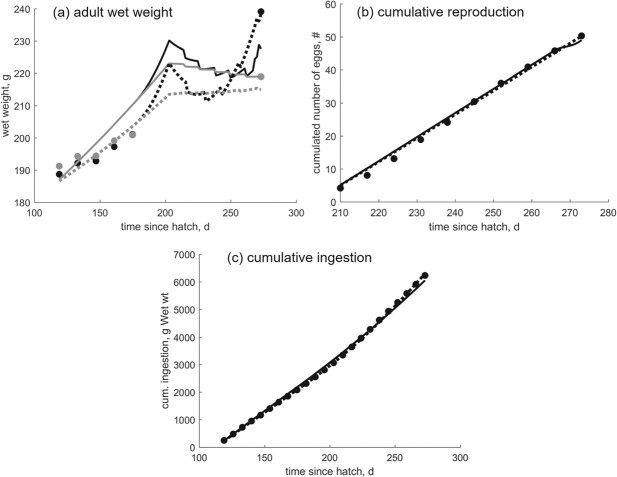
Model predictions for wet weight, cumulative egg production and ingestion of adult quail in Bayer data report M-299245-02-1 (Bayer, 2008). In panel (a), black color refers to females and gray color to males. Model predictions obtained with Hypothesis 1 for energy allocation are plotted as dotted lines, and model predictions obtained with Hypothesis 2 as full lines. Relevant parameters for model predictions are given in Table 2. The wet weight of the reproduction buffer is included in the total wet weight calculation.

We combine the mean values from the report with data available in literature to estimate the parameters of the DEB quail model (data and corresponding model predictions are presented in Table 3 and Figs 2 and 3). The list of estimated parameters is presented in Table 2. Parameter estimation was based on standard approaches and tools used for DEB models: parameters were estimated by minimizing a loss function, which generally is a function of data, predictions, and weight coefficients (Marques *et al.*, 2019). We chose the symmetric-bound loss function, and the minimization was done by the Nelder–Mead simplex method (Marques *et al.*, 2019). The estimation of the parameters builds on the existing standard DEB model entry in the AmP collection (AmP *Colinus virginianus*, version 2017/08/09, AmP, 2021) and was performed in Matlab using routines freely available in the software package DEBtool (DEBtool, 2021, Lika *et al.*, 2011, Marques *et al.*, 2019). Two goodness-of-fit measures were used to evaluate the overall model performance: the mean relative error (MRE) and the symmetric mean squared error (SMSE) (Marques *et al.*, 2019).

**Table 3:** Zero-variate data used to calibrate the model, the corresponding model predictions, and the relative error as percent in brackets

Symbol (units)	Data	Prediction (RE)	Prediction (RE)	Description	Source
		(H1)	(H2)		
$a_b$ (d)	23	23.56 (2.4%)	20.3 (11.7%)	age at hatching	(1)
$t_p$ (d)	100	105.1 (5.3%)	110.8 (10.7%)	time since hatch at puberty	(2,3)
$a_m$ (d)	2336	2340 (0.2%)	2327 (0.4%)	life span	(4)
$L_i$ (cm)	26	26.0 (0%)	25.64 (1.4%)	ultimate total length	(5)
$W_{wb}$ (g)	6.5	6.53 (0.5%)	6.72 (3.5%)	wet weight at hatch	(6)
$W_{w14}$ (g)	34.37	34.31 (0.2%)	35.35 (2.8%)	wet weight at 14 days since hatch	(7)
$W_{wx}$ (g)	182.9	182.4 (0.3%)	183.3 (0.2%)	wet weight at fledging	(8)
$W_{wp}$ (g)	194.2	182.8 (5.9%)	183.9 (5.4%)	wet weight at puberty	(8)
$W_{wi}$ (g)	200	202.3 (1.1%)	209.2 (4.6%)	ultimate wet weight	(7)

Data sources: (1) Hernandez & Peterson (2007); (2) Schom & Abbott (1974); (3) Flores-Santin *et al.* (2018); (4) Zammuto (1986); (5) Shanaway (1994): (6) Newsted *et al.* (2008); (7) Bayer (2008); (8) Robel & Linderman (1966).

The two hypotheses for energy allocation were evaluated qualitatively. We compared the match between the data and corresponding model predictions obtained by each of the two hypothesis. The predictions were evaluated also in the context of mother-offspring link, as this information was available in the study report. Finally, we calculate the respiration as predicted by the model under each hypothesis, and compare it with the data available in a study performed on a closely related species, the Japanese quail (Pick *et al.*, 2016).

#### Variability in data

After parameterizing the model and selecting a more likely hypothesis (pathway) for energy allocation, we use the model which includes the selected hypothesis to reproduce the observed variability in the data available in the scientific report on the quail OECD reproduction study (Bayer, 2008, control). The data show considerable variability in number of eggs per cage, variability of weight at hatch (implying variability in egg size), and variability in weight of adults and 14-day-old chicks. During the 10 weeks of egg-laying, the number of eggs per week differ within and between cages, without a discernible pattern in egg-laying throughout time (Bayer, 2008). Egg weight data were not available in the report, so we assumed a strong correlation between wet weight of chicks at hatching (data available in the report) and the egg weight. This was confirmed by data from another study (Bayer, 2004), where egg mass and hatchling body mass indeed correlate ($r=0.956$) and have similar variability ($CV$ of about 7%). Variability in weight at hatch within cages is up to 15% $CV$ in some cages (mean $CV$ 8.45%), 14-day-old chick weight shows variability within and between cages, as does the adult weight (males and females). We explored potential sources of the observed variability by modifying selected model parameters and analyzing resulting patterns, as explained in the following section. We assumed three main sources of scatter: (i) time between egg-laying, (ii) egg size, and (iii) physiological inter-individual variability.

(i) To generate scatter in time between egg-laying, $t_e$, random numbers were generated from an exponential distribution with mean $1/\dot {k}_R$ = 1.32 d, where $\dot {k}_R$ is the rate of egg-laying (estimated from data). Intervals longer than 10 days were omitted from analysis and excluded from simulations, because these events were relatively rare and were as such considered outliers, and likely an artifact of the way the rate of egg-laying was reported, i.e. only at an end of each week.

(ii) Variability in egg size was introduced by adding scatter to the value $E_0$ obtained by assuming the maternal effect (DEB implied property). Stochasticity was introduced to reflect the fact that wet weight of chicks (and therefore the corresponding initial egg sizes) vary within and between cages, irrespective of the week of the experiment. Values for egg size were randomly drawn from a normal distribution, with mean being the value $E_0$ (calculated via maternal effect rule) and coefficient of variation $CV = 8.45\%$ (a mean value calculated from variability in weight at hatch). Generated values smaller than $E_0^{\mbox {\small {min}}}$ or larger than $ E_0^{\mbox {\small {max}}}$ were omitted, where $E_0^{\mbox {\small {min}}}$ and $E_0^{\mbox {\small {max}}}$ are calculated based on the smallest and largest observed egg (from data, Smith *et al.*, 1996). The $E_0$ itself will vary in time because of variable feeding, which implies a non-constant $[E]$ and, consequently, non-constant $[E_b]$ (maternal effect assumption). This, however, cannot explain the variability in weight at hatch (and therefore the corresponding initial egg sizes) within the same week, because short-term changes in feeding are smoothed by the reserve buffer.

(iii) Finally, to reproduce observed inter-individual variability among adult hens in wet weight and ingestion, and among juveniles in the wet weight they attained after 14 days, we introduced inter-individual physiological variability. This was done assuming that parameter values related to the physiological design of the organism vary between individuals (with small variation, here $CV =1\%$) via the zoom factor $z = L_m/L_m^{ref}$ according to the covariation rules. Here, $L_m = \kappa \{\dot {p}_{Am}\} /[\dot {p}_M]$ is the maximum structural length and $L_m^{ref} = 1$ cm is a reference maximum structural length. Effectively, we introduced variability in the maximum surface area-specific assimilation; individuals relate to the ‘average’ individual as $\{\dot {p}_{Am}\} = \frac {z}{z_0} \{\dot {p}_{Am}^0\}$, where $z^0$ and $\{\dot {p}_{Am}^0\}$ are, respectively, the zoom factor and the maximum surface area-specific assimilation of the ‘average’ individual, estimated by the DEBtool routines. Individuals were assigned different values of the zoom factor $z$ sampled randomly from a normal distribution with mean $z_0$ and standard deviation $\sigma $ ($=CV \cdot z^0$). As a result, simulated individuals will differ in their assimilation potential and growth potential, resulting in different growth and reproduction patterns.

We simulated 25 individual hens, and their respective offspring. Simulations were performed for 186 days after puberty; this includes 67 days of pre-photostimulation, 40 days of photostimulation and 10 weeks of egg-laying period. All simulations were done in Matlab.

## 3 Results

DEB model formulation for both hypotheses (H1 and H2) resulted in predictions that match well the various types of data used for model parameterization, i.e. overall goodness of fit statistics are somewhat better for H1, but are generally similar for both hypotheses: 0.022 MRE and 0.028 SMSE for H1 and 0.045 MRE and 0.051 SMSE for H2. We first present, for both hypotheses, model parameters and fit of model predictions to data used for model parameterization. In the next step, we reject H1 (that the extra assimilates are directly allocated to egg production) and select H2 (that the extra assimilates are first added to the reserve) as the more likely hypothesis for energy allocation. We then present data variability simulations for H2.

**Model parameters.** Estimation of model parameters was based on the previous AmP entry version (AmP *Colinus virginianus*, version 2017/08/09, AmP, 2021) as initial parameter values, and produced realistic values for both energy allocation hypotheses (H1 and H2, Table 2). Compared with the initial parameter set having a relatively high value of the volume-specific maintenance rate $[\dot {p}_M] = 2150$ J/d.cm$^3$ and the surface-area specific assimilation rate $\{\dot {p}_{Am}\} = 4940$ J/d.cm$^2$ (AmP, 2021), the lower volume-specific somatic maintenance of $[p_M] \approx $ 200 J/d.cm$^3$ is a more realistic value, paired with $\{\dot {p}_{Am}\} \approx 1300$ J/d.cm$^2$. In more practical terms, the model with, the H2 hypothesis parameter set predicts that a fully grown individual experiencing abundant food can assimilate roughly 9200 J/day, and needs to pay roughly 6000 J/day to cover maintenance costs (3650 J/day of somatic maintenance plus 2400 J/day of maturity maintenance), leaving >3000 J/day available for other processes. Predictions with the initial parameter set (AmP, 2021), meanwhile, suggested that a fully grown adult can assimilate 18700 J/day, but needs 17500 J/day to cover maintenance costs (15800 J/day + 1700 J/day), leaving a bit over 1000 J/day for other processes. The difference between the assimilated energy and that required for maintenance is a good indicator not only of the energy available for maturation and reproduction (Fig. 1), but also of the individual’s potential to have excess energy and store it for later, which would be helpful in times of food shortages. If not enough energy can be mobilized from reserve to cover the cost(s) of maintenance, the structure *can* be re-used and the organism shrinks. The cost of building structure is estimated at roughly 7300 J/cm$^3$ (Table 2), so the energy gained by re-using that structure would be 5850 J/cm$^3$ (roughly 80% of energy invested into building a unit of structure; see Section 4.1.5 comments to Kooijman, 2010; Kooijman, 2022). If the costs of maintenance are lower, then the required level of shrinking during food shortages is also lower, which is a preferred option. The drastically lower value estimated for $[\dot {p}_M]$ also enables the adult to grow to a relatively larger size (ultimate structural length of 2.57 cm compared with 1.94 cm predicted with the initial parameter set).

Another major difference in parameter values is the value of parameter $\kappa $. This parameter determines the proportion of the mobilized energy allocated to somatic maintenance and growth, and had a value of 0.84 in the initial parameter set (AmP, 2021). The newly estimated value of $\kappa =$ 0.49 (H1) and 0.39 (H2) (Table 2) implies that less than half of the mobilized energy is allocated for somatic maintenance and growth, while the remainder is available for maturation and reproduction (see Fig. 1 for a schematic representation of energy fluxes). Such a low value of $\kappa $ makes sense for birds reared for egg-laying, but might be different for birds in the wild.

It was possible to estimate the assimilation efficiency $\kappa _X$ by using data on ingestion, and this produced a realistic value of around 0.5, matching the value estimated for other birds. The proxy for food availability — scaled functional response $f$—was for young juveniles estimated higher than the reference $f=1$, implying food of higher quality or quantity than that experienced by adults, or higher digestibility of the same food. The estimated values $f>1$ reproduced the fast growth reported for juveniles (Fig. 2c and d).

**Fit of model predictions to data used for model parameterization**

Model predictions match well the data for basic life events (hatching, fledging, puberty, life span) and corresponding size of a generalized northern bobwhite quail, regardless of the energy allocation hypothesis used to obtain model predictions (Table 3). Predictions for wet mass and length of embryo, wet mass of juveniles and adults, and cumulative ingestion and egg production of adults all match the data extremely well, and are practically indistinguishable between the two hypothesis (Figs 2 and 3). For embryos and juveniles, (i.e. individuals that do not reproduce), predictions obtained with H1 and H2 models slightly differ due to different parameter values (see Table 2 for parameter values and Fig. 2 for model prediction). For reproducing adults wet weight fluctuations will differ due to different energy (and mass) pathways, even though models for both hypotheses result in increased food intake, corresponding growth in wet mass, and egg production (Fig. 3). The predicted wet weight fluctuations are, to a large extent, a result of the filling and emptying of the reproductive buffer, the dynamics of which depend on the hypothesis used to obtain the predictions. The wet weight was calculated by adding the weight of structure, reserve, and the reproductive buffer; the hen was increasing her weight by eating, and decreasing it by laying eggs. Weight fluctuations are more pronounced for H1 predictions, because of the relative contribution of reserve $E$ and reproductive buffer $E_R$ to the total wet weight. During egg-laying, the reproductive buffer increases and the reserve decreases for H1. The opposite happens for H2, where reserve contribution to wet weight is larger than that of the reproductive buffer. Unfortunately, weight data for the actual egg-laying period are not collected in OECD 206 studies in order to avoid hen disturbance (OECD, 1984), so there is no easy way to see which predictions are more in lines with what we observe.

**Figure 4:**
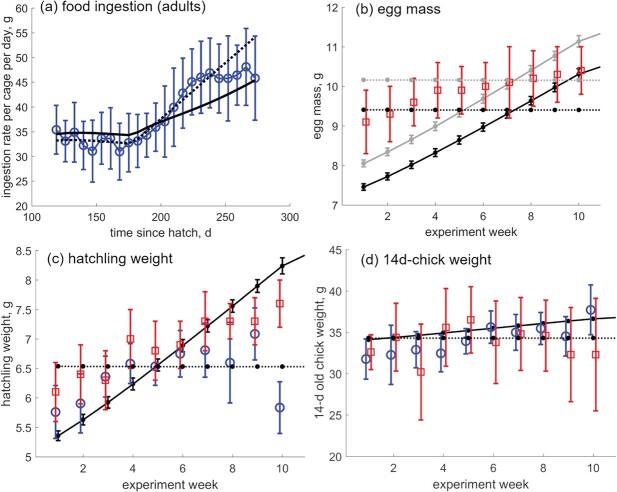
(a) Food ingestion rates of adults, expressed per cage data from Bayer (2008): observations are marked with blue circles (mean $\pm $ sd) and predictions with lines (dotted line, H1; solid line, H2). (b–d) Wet mass of eggs, hatchlings and 14–day-old chicks: data are marked as blue circles (Bayer, 2008) or red squares (Bayer, 2004) as mean $\pm $ sd, and predictions are plotted with black lines (dotted line, H1; solid line, H2). In (b, egg mass) the gray lines denote the predicted egg mass increased by 8% (Yannakopoulos & Tserveni-Gousi, 1986); see Discussion for details. Mean $\pm $ sd of predictions is marked with dots to aid comparison. See Fig. 1 for hypotheses scheme.

**Hypothesis 1 or Hypothesis 2?** Based on the results presented thus far, it would be extremely difficult to determine with certainty which hypothesis for energy allocation is more plausible, with H1 resulting in a slightly better fit of model predictions to data. An important aspect to note is that so far all of the presented data was used to estimate model parameters, and that the data was collected from different sources but approximate a general average individual from fertilization (age 0), to adulthood, reproduction, and death. In our next steps, we present data either in a different way (ingestion rate per cage per day instead of the average cumulative ingestion, cf. Figs 4a and 3c), or we use all of the average weekly values instead of the average value in the experiment (cf. Fig. 4b–d and Table 3). Additionally, we were able to directly link the condition of the mother to the size and growth of the offspring, as the information about which offspring comes from which mother is explicitly reported in the reproduction study report (Bayer, 2008). Food ingestion was back-calculated based on the estimated value for scaled functional response $f$, using the estimated value for assimilation efficiency $\kappa _X$ (Table 2).

Focusing on adults only, either hypothesis H1 or H2, could again be correct: both energetic pathways will result in an increased food ingestion (Fig. 4a). H2, however, where females increase feeding to compensate for increased maintenance costs during the reproductive phase, is more in line with the observations for offspring: the average predicted egg mass, hatchling mass, and 14-day-old chicks mass is increasing as reproduction progresses (Fig. 4b–d).

Final piece of evidence in favor of H2 is the prediction that the increased metabolic costs result in 9%–25% higher O$_2$ consumption (non-feeding and feeding hens, respectively, Table 4), which is lower than the 60%–70% increase of resting metabolic rate (RMR) reported for breeding Japanese quail (Pick *et al.*, 2016), but in accordance with the 16%%–24% increase of RMR presented in the review on passerine birds (Vezina & Salvante, 2010). By contrast, the consequence of H1 would be an approximately 20% *decrease* in $O_2$ consumption (non-feeding and feeding hens, respectively, Table 4). Therefore, H1 was rejected, and H2 was incorporated into the model for the next step of data variability analysis.

**Table 4:** Predictions for metabolic rates obtained by using the two hypothesis (H1 and H2) for energy utilization during reproduction. The predictions are compared$^{a}$ to the values obtained from the study by Pick *et al.* (2016) on a related Japanese quail. Pick *et al.* (2016) report that food was withdrawn 2–3 hours before the metabolic rate was measured; we report values with$^{1}$ and without feeding$^{2}$ because some contribution of feeding to respiration is unavoidable.

	Hypothesis 1 (H1)	Hypothesis 2 (H2)	Pick *et al.* (2016)
	*Start of experiment*		*Non-breeding*
Wet weight (g)	186.56	186.88	240 $\pm $ 17
	*End of experiment*		*Breeding*
	238.32	227.48	254 $\pm $ 20
Relative change in weight-specific			
O$_2$ consumption$^1$	-23.61%	25.29%	–
O$_2$ consumption$^2$	-20.97%	9.22%	61.2%

$^a$ To directly compare values, we focus on the relative change in the mass-specific respiration. We use the average respiration (expressed in ml O$_2$/min) and wet weight of hens (g) reported in Pick *et al.* (2016) to calculate the relative difference in mass-specific O$_2$ consumption between breeding and non-breeding Japanese quail. For our model predictions, we use the weight at the beginning and end of the experiment to approximate non-breeding and breeding birds (respectively), taking into account that there is not much weight change in adult birds if they are not stimulated to reproduce (Flores-Santin *et al.*, 2018). The model predicts respiration in mmol O$_2$/g/d, and we use this to calculate mass-specific respiration, and then the relative change.$^1$With feeding; $^2$No feeding

**Figure 5:**
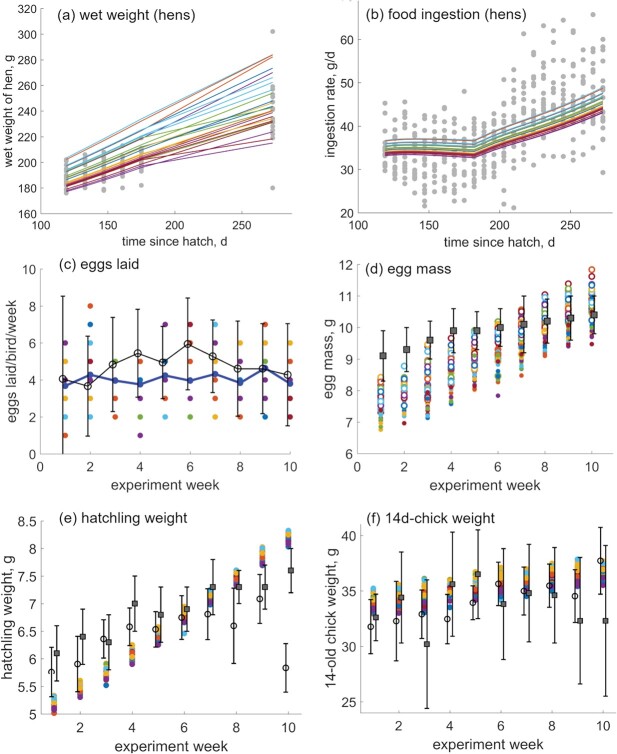
Monte Carlo simulation (25 hens) with simultaneous variability in egg-laying rate, egg size and specific maximum assimilation efficiency (see text for details). Predictions are plotted in color and data are plotted in grayscale. (a and b) Wet mass and food ingestion of adults throughout the experiment; dots represent data from Bayer (2008), lines predictions. (c) Eggs laid per bird per week mean; black empty circles ($\pm $ sd) represent data from Bayer (2008), colored dots with means in blue points are predictions. (d–f) Wet mass of eggs, hatchlings and 14–day-old chicks; black empty circles and full squares squares represent data (mean $\pm $ sd) from Bayer (2008)(circles) and Bayer (2004) (squares), colored dots are predictions. All predictions obtained with Hypothesis 2.

**Simulations for data variability** We simulated 25 individuals, simultaneously differing in (i) time between egg-laying, (ii) size of eggs they produce, and (iii) physiological characteristics modeled via DEB parameter $\{\dot {p}_{Am}\}$, using Hypothesis 2 for energy allocation. Model outputs for 25 simulated individuals are in Fig. 5. Overall, the model was able to reproduce the variability in both adults and chicks, and the ranges of values predicted in the simulations matched those observed for egg mass, wet weight at hatching (birth) and wet weight at 14 days (Fig. 5). The model simulations suggest that the hens occasionally enter the condition of starvation, despite the increased food ingestion; we discuss this later in the context of energy budgets.

Because of the model setup, all three sources of variability act in a complementary way: (i) egg-laying rate has the most direct effect on the number of eggs laid per week (Fig. 5c), but it also affects the wet weight of adults: the more frequent is the egg-laying, the faster is the emptying of the reproductive buffer and the smaller its contribution to wet weight. The mean time between laying eggs is 1.43 days, i.e. the data-driven egg- laying rate (estimated as $\dot {k}_R = 0.7$ eggs per day) results in on average 3.976 eggs per week, which is slightly lower than the average observed values (Fig. 5c) and the overall observed average of 5 eggs per week (Bayer, 2008).

(ii) Egg size variability shapes directly the predictions for egg weight and the subsequent hatchling and 14-day chick weight (Fig. 5d–f), but will also affect the wet weight of adults during the egg-laying period (Fig. 5a). This is because the reproduction buffer is emptied by laying eggs, and so the larger the egg, the more energy is removed from the reproductive buffer by each egg.

(iii) Different maximum assimilation efficiency $\{\dot {p}_{Am}\}$ results in different amounts of energy available for growth and reproduction. A direct result is a range of sizes, comparable with data via predicted weights (Fig. 5a) and food ingestion (Fig. 5b), which is calculated as proportional to the hens surface area of structure only (*not* directly proportional to wet weight). Wet weight is calculated by summing up reserve, structure and reproductive material of the hen. Even though the range of predicted values is matching the data well, the values for the last data point are slightly larger than observations. For food ingestion, by contrast, the predictions match the observed values; however, the range of predicted values is much smaller than that of the observations (Fig. 5b). We come back to these two points in the discussion.

## 4 Discussion

We focused on the bobwhite quail — a well studied and widely distributed species — for which we present a full life cycle mechanistic model. The model is a standard DEB model extended with an egg-laying module, and we have shown that it can predict well all aspects of the quail’s life cycle and reproduction. We identified Hypothesis 2 (H2) — where extra energy ingested prior to and during egg-laying is first assimilated into reserve and then used for other processes, including reproduction — as the more likely energy pathway for the extra assimilates. The predictions for this hypothesis result in increased metabolic costs and respiration, matching the literature (Pick *et al.*, 2016, Vezina & Salvante, 2010). Then, using the model based on H2 and a few selected parameters as sources of variability, we reproduce the observed data variability remarkably well.

### Assessment of model fit (H2) and assumptions

The predicted age at hatch of around 20 days matches the average incubation duration of 23 days at 37.5 $^{\circ }$C–38.5 $^{\circ }$C (Green & Vince, 1973, Kato *et al.*, 2013, 2014, Reyna, 2019, Reyna & Burggren, 2017, Walter & Voitle, 1973). Predicted wet weight at hatch of 6.7 g is only 3.5% larger compared with the data used for parameterizing the model (Table 3) and is close to the average hatching mass (6–6.5 g; Brennan *et al.*, 2020, Walter & Voitle, 1973). Predicted egg size and simulated variability in egg size and hatchling mass generally match the variability observed in the data (Fig. 5d–f). The non-corrected predictions for egg mass (average 8.7 g) were lower than observations, and include a wider range and lower values than observations (black lines in Fig. 4b and full dots in Fig. 5d). However, the predictions were improved after accounting for material that cannot be mobilized during development and growth, such as shell plus extraembryonic membranes, which comprise on average 7.8% of egg weight (Yannakopoulos & Tserveni-Gousi, 1986). Increasing the egg mass predictions by 8% resulted with the average egg mass of 9.44 g, matching the data (gray lines in Fig. 4b and empty dots in Fig. 5d) and the literature: eggs of flight-type bobwhites were found to, on average, weigh 9.62 g (range 9.34–9.97 g) (Walter & Voitle, 1973), 9.3–9.8 g (Lyon, 1962) and 10.4 g (range 8.8–11.6 g) (Smith *et al.*, 1996). These predictions, together with a well-predicted embryo growth (Fig. 2), imply that the embryonic development is modeled consistently and well. This satisfies one of the major objectives of the study: to develop a precise quail model, which also enables testing various further scenarios. For example, the quail embryo development during the final few days prior to hatching is analogous to that of other similar species (chicken, fowl, Japanese quail) (Green & Vince, 1973), so the quail model can be used to obtain relevant predictions for other species.

Growth of juveniles was modeled by assuming a non-constant food assimilation for the early juvenile period (50–60 days), which was reflected as a larger-than-reference $f>1$ parameter for food availability. The predictions matched well the data from the literature (Jones & Hughes, 1978, Lyon, 1962, Newsted *et al.*, 2008; see Fig. 2). Individuals will eat food of different quality throughout life, or of different relative quantity (relative to their body size) (Brennan *et al.*, 2020). Therefore, the food level of an individual quail is generally not constant throughout its life. Different (compared with reference) (i) quality of food, (ii) digestibility of food (either due to food itself or physiology of an individual), or (iii) ingestion rate of food, can all result in values of the scaled functional response $f$ that deviate from the reference value of 1. Individuals experience a higher-than-reference $f$ in their early juvenile period, and again in their reproduction period. As a consequence, the early juveniles will grow faster than they would at a reference food level ($f=1$), and the adults will be able to produce more eggs and will gain more weight during the reproduction phase, which is in accordance with observations.

It is intuitively easy to grasp that the main reason for different-to-reference-growth lies in the food, because we know that food changes throughout ontogeny (Brennan *et al.*, 2020). However, the first 2 weeks of chick life are extremely physiologically eventful, as thermoregulation is initiated, feathers begin to grow, and flight and walk muscles develop: as precocial birds, little quails can walk almost immediately upon hatching and can take their first flight usually about 14 days after hatching (Brennan *et al.*, 2020). These physiological changes all have associated metabolic costs, even in rearing facilities, and are currently considered a small part of the general somatic maintenance and are included in the parameter [$\dot {p}_M$] (Kooijman, 2010). However, such changes might influence the shape of the growth curve, especially for wild young quail, and can be modeled explicitly if enough information is available (e.g. Jusup *et al.*, 2014). Including the cost of thermoregulation into the model — not only for chicks but also for hens as part of parental care (Beekman *et al.*, 2019) — might be very relevant when adjusting the model for wild quail (or other bird) populations.

Size and age at puberty, as well as adult size and reproductive output are also predicted extremely well (Table 3, Figs. 3 and 5a,b). This is crucial in the context of (i) definition of maturity (puberty) as ‘reaching adult weight’ *or* ‘beginning of egg laying’, because the two events can be quite some time apart (Flores-Santin *et al.*, 2018, Schom *et al.*, 1979, Schom & Abbott, 1974); (ii) adult weight varying across the species’ geographic range and fluctuating depending on the reproductive activity (Brennan *et al.*, 2020, Cain & Lien, 1985, Schom *et al.*, 1979); and (iii) egg-laying rates and total reproductive output varying extensively not only between wild and captive-reared birds, but also among captive-reared quails (Baldini *et al.*, 1952, Brennan *et al.*, 2020, Quinn *et al.*, 2009, Reyna & Burggren, 2017, Schom & Abbott, 1974, Valverde-Garcia *et al.*, 2018). De-coupling (the energetic requirements for) maturation from that for reproduction, and de-coupling growth from maturation/reproduction, enable linking certain environmental conditions, such as temperature and food availability, to expected size and reproductive output of the individual (see, e.g. Marn *et al.*, 2017). This is especially relevant for species that can have a prolonged period between puberty and onset of reproduction, as well as species that exhibit a large variability in size and reproductive output; even more so in the context of climate change. External sources of variability can be reproduced by the right mechanistic model with the well-chosen forcing variables, and can be complemented with internal (physiological) sources of variability, as we have done in this study for the captive reared quail.

The de-coupling of puberty from certain size and age is possible due to the model setup: the DEB model tracks maturation separately from growth, so ‘puberty’ is defined as reaching a maturity threshold of energy invested into maturation, rather than attaining a certain size or age (Kooijman, 2010). Estimating or calibrating the value of the maturity ‘puberty’ threshold, however, *does* require associating a certain size (weight and/or length) and age to the transition from juvenile to adult stage. The earliest ‘age at maturity’ mentioned for the bobwhite quail in the literature, where ‘maturity’ was defined as reaching the adult body weight of $\approx $200 g, is 100 days post-hatching for quails reared at abundant food and constant 12-hour-light days (Flores-Santin *et al.*, 2018). Therefore, values of 100 days and 200 g were used as age and size at puberty for parameterizing the model at reference food and temperature (Table 3). A higher age at puberty could be possible for different rearing conditions (Lyon, 1962, Schom *et al.*, 1979), and especially for the wild populations, where individuals do not reproduce before spring of the following year when they are almost 1 year old (Baldini *et al.*, 1952, Brennan *et al.*, 2020).

Differentiating between juveniles and non-reproducing adults can be done based on plumage (Brennan *et al.*, 2020, Lyon, 1962), but most often adulthood is determined via wet weight. This is because, as a general rule, weight fluctuations occurring after gaining the ‘adult weight’ can generally be linked to reproductive cycles, not additional growth or maturation. For example, individuals kept at constant 12- or 6–8-light days do not exert significant wet weight fluctuation after attaining the ‘adult’ or ‘mature weight’ (Fergin & Schafer, 1977, Flores-Santin *et al.*, 2018), but a notable increase in wet weight is observed after light stimulation even in older birds: for example, 2-year-old quails increased their weight by 15% in 112 days (Schom *et al.*, 1979). A weight increase results both from increased food consumption as well as the expansion of the reproductive system (Fergin & Schafer, 1977), and if egg-laying birds are deprived of some food and water, they start losing 4%–8% of their weight and cease to produce eggs (Cain & Lien, 1985). However, despite ‘adult weight’ being a convenient way to determine adulthood, we cannot exclude the possibility that birds are ‘mature’ even before attaining the ‘adult weight’, especially if food conditions are poorer and birds are leaner. Such scenarios of lower food availability, possibly as a result of climate change or anthropogenic pressures, can be explored by a mechanistic model such as the one presented in this study.

Attaining adulthood is not the only pre-requisite for reproduction: stimulation of the pituitary gland — induced by long-light days and resulting in recrudescence of reproductive organs — needs to occur, combined with sufficient food availability (Brennan *et al.*, 2020, Cain & Lien, 1985, Nordstrom, 1973, Ottinger *et al.*, 2005, Vezina & Salvante, 2010). Typically 30–40 days are reported between light stimulation and onset of reproduction in all standard quail studies (Schom & Abbott, 1974, Valverde-Garcia *et al.*, 2018), and when light cycles are used to synchronize breeding couples of different ages, again usually 30–40 long-light days are needed for females to start laying eggs (Schom & Abbott, 1974). The observations suggest that 30–40-day period with long-light (and high food availability) is needed either to prepare the reproductive system, and/or to accumulate energy for eggs. Therefore, we included a period of 40 days between puberty and onset of reproduction into the model. During this period, the food ingestion was estimated to be higher compared with the reference food ingestion, which matched the observations and consequently resulted in overall increased wet weight and egg production, also matching the data (Fig 3). Interestingly, even with the extra food available *ad libitum*, the period of reproduction is so energetically demanding that, according to our simulations, the hens occasionally may enter the condition of starvation. The likely explanation is that attaining adulthood is coinciding with attaining the ultimate size, at which point the input (energy assimilation) and output (energy mobilization to cover maintenance costs) are balanced. This makes the associated costs of reproduction hard to satisfy, and explains why the extra food intake is necessary as preparation (not only as a consequence) of reproduction. Currently, the length of this period (40 days) is set manually and is driven by the experimental setup, not by the condition of the individual. However, because the model design is modular, this step can be refined with sufficient knowledge and data (see, e.g. Muller *et al.*, 2019).

Hens that can be induced by long-light days to reproduce are often referred to as ‘immature adults’, but are considered ‘full’ adults in DEB terms. It would be interesting to empirically quantify energy fluxes during the period between puberty and reproduction, as the standard DEB model (applied here) implies that certain amount of energy is allocated to reproduction even during this stage. This is especially relevant for wild birds, which have seasonal food availability and reproduce only once or twice per year, and theoretically allocate to reproduction throughout the year (but see Muller *et al.*, 2019). The buildup (recrudescence) and subsequent atrophication of the reproductive organs are especially important from the energetic perspective, as they incur substantial metabolic activity (Pick *et al.*, 2016, Vezina & Salvante, 2010), which can be modeled within the presented framework (Kooijman, 2010, Muller *et al.*, 2019 and Kooijman, 2022, comments to DEB3). Quantifying these processes further might point to potential physiological differences between wild and reared birds, showing up as differences in relevant DEB parameter values (see, for example, entries for *Gallus gallus* available at http://www.bio.vu.nl/thb/deb/deblab/add_my_pet/entries_web, and some other examples already present in the AmP collection, AmP, 2021).

### Modeling inter-individual variability

Performance and applicability of a mechanistic model could be evaluated not only by how well it fits the average life cycle events and particular growth or reproduction curve for a species, but also how well it can reproduce the observed variability. The aspect of data variability is especially important when parameterizing a model for an average individual, with the aim to make biologically and ecologically relevant predictions for multiple individuals and eventually populations. Even in a standardized study performed under controlled laboratory conditions (Bayer, 2008), there was considerable variability in all measured data: adult mass, food ingestion, eggs laid per week, hatchling mass and growth. We managed to reproduce the variability to a satisfactory extent (Fig. 5) by making explicit assumptions and introducing only three main sources of variability: egg-laying rate, egg size, and assimilation potential (physiological variability).

Egg-laying rate, $\dot {k}_R$, is conceptually a new DEB parameter determining the time between laying two eggs. Physiologically, this would translate to a different time span required for each oocyte to finalize yolk deposition, then ovulate and move through the oviduct, resulting in varying number of eggs laid each week, which is what we observe in data (Fig. 5c). Generally, the $\dot {k}_R$ value might differ for wild quails, which have different egg-laying dynamics compared with the reared ones (Alisauskas & Ankney, 1994, Brennan *et al.*, 2020, Piccirillo & Orlando, 1985, Quinn *et al.*, 2009). Within this model setup, the explanation for a different egg-laying rate might be external or internal. For example, seasonal food availability and natural day–night cycles will act as external drivers. Energy allocation pathways, namely the proportion of mobilized energy allocated to reproduction — quantified as (1-$\kappa $) — is an example of an internal driver for the egg-laying rate. Of Galiiformes in AmP, all have a higher $\kappa>0.8$ except two chicken strands reared for egg-laying, with $\kappa <=0.5$ (AmP, 2021). This could imply a physiological difference between the wild type and those bred for egg-laying. It has also been noted that the egg-laying rate will have big implications on the energy budget of the hen (Alisauskas & Ankney, 1994), but this has not been explicitly linked to the up-regulation of feeding or specific physiological characteristics.

**Figure 6:**
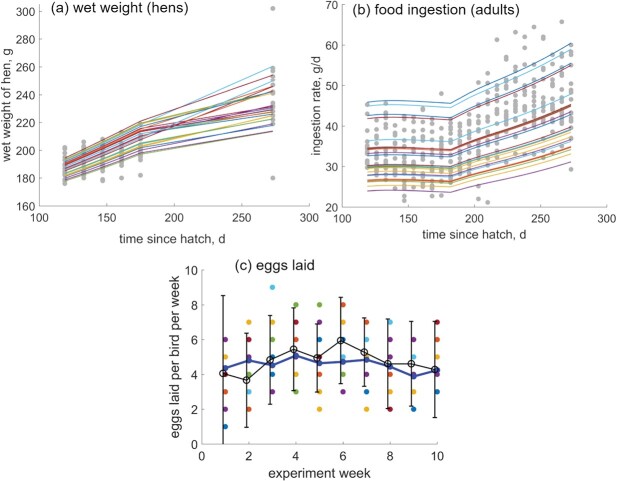
Monte Carlo simulation (25 adults) with variability in egg-laying rate, egg size, and inter-individual variability affecting both food ingestion ($\kappa _X$) and food assimilation ($\{\dot {p}_{Am}\}$) (see text for details). Predictions are plotted in color and data are plotted in grayscale. Top panel: Predictions for wet mass and food ingestion of adults throughout the experiment; dots represent data; lines predictions. Bottom panel: mean ($\pm $ sd) observed eggs laid per bird per week (black); predicted egg laid per bird per week (colored dots with means in blue points). The mean time between laying eggs is approximately 1 day, which results in on average 4.552 eggs per week. Predictions obtained with Hypothesis 2.

Even though the range of eggs laid per bird per week is predicted well, as is the cumulative egg production (Fig. 3c), the average number of eggs laid per bird per week is under-predicted (Fig. 5c). In the context of hen mass being over-predicted (Fig. 5a), this suggests that in the simulations too much reproductive material is staying in the hen. A likely ‘culprit’ is the aforementioned egg-laying rate $\dot {k}_R$. The parameter value $\dot {k}_R \approx 0.7$ d$^{-1}$ has been estimated from (laboratory) data that was reported per week—some information about daily egg-laying rates is therefore lost—and then expressed as cumulative average number of eggs for parameter estimation (Fig. 3b), thereby further removing the fine details. If we fit the exponential distribution to egg-laying data for individual cages, we obtain means for the $\dot {k}_R$ parameter ranging from 0.25 d$^{-1}$ to 1.05 d$^{-1}$, i.e. average time between laying two eggs ranging from 0.95 d to 3.94 d. We repeated the Monte Carlo simulations with values for $\dot {k}_R$ in this range to see if we can reduce the mis-match between data and predictions. We managed to reproduce the range and means of observed values for eggs laid per week (see Fig. 6b) with $\dot {k}_R=1$ d$^{-1}$, incidentally matching the egg-laying rate of the wild quail (Brennan *et al.*, 2020, Reyna & Burggren, 2017). Model simulations now matched data for both the adult wet weight and egg-laying rate (Fig. 6a,c).

To reproduce the observed range in ingestion rates, we generated variation in digestion efficiency. Motivation for introducing variability in digestion efficiency was two-fold: (i) it is the most logical and the simplest way to increase the range of predicted ingestion rate values for individuals of a certain size range because of the way the food ingestion is calculated (Kooijman, 2010), and (ii) some food might have been spilled during the experiment (Diana Temple, pers. comm.), which is mathematically translated into lower digestibility of the food. Generally, data on food ingestion can be used directly as a model forcing variable if enough information is provided. Unfortunately, only weekly data on food usage per cage was available, with sources of uncertainty both in possible food spillage and in uneven food consumption of females relative to males (Diana Temple and Colin Scanes, pers. comm.). The data, however, *did* enable comparison of model simulations with the observations, i.e. theoretical model exploration. We generated variation in digestion efficiency by drawing numbers from a beta distribution with shape parameters $a=20$ and $b=19$, i.e. the mean of distribution is 0.505 (estimated value for $\kappa _X$, see Table 2) and variance 0.006. This produced predictions matching the data very well (Fig. 6b).

The current implementation of the egg-laying module was based on data-analysis and specific modeling assumptions rooted in information available for the bobwhite quail and birds in general. We have considered extending this part of the egg-laying module to mechanistically model the oocyte maturation and release (see, for example, Muller *et al.*, 2019). This would be done by ‘preparing’ and tracking several oocytes simultaneously and then releasing them as the egg size $E_0$ is attained, mimicking what we observe in the chicken: during maturation, a hierarchy of maturing follicles will develop so as to supply a sequence of eggs for daily ovulation (Nordstrom, 1973, The VetMed webpage, 2019). A possible benefit would be that we can follow the egg while still in the mother, even though one can (arguably) do this already with the current egg-laying module. The biggest obstacle is lack of data. For example, in the mature (White Leghorn) hen, the ovary at any given time has 3–4 large ‘maturing’ follicles, and a series of 8–12 follicles of diminishing size (Nordstrom, 1973, The VetMed webpage, 2019). In a quail-related species (ruddy ducks) there are never more than 5–6 rapidly developing follicles in any female (Alisauskas & Ankney, 1994). We do not have information on number of oocytes maturing simultaneously in quail ovary, but could possibly use the ovary weight ratio with some associated uncertainty and error: in the mature hen the ovary should weigh around 35 g (The VetMed webpage, 2019) whereas the mean ovary weight of reproducing bobwhite is around 4.5 g (Cain & Lien, 1985). However, no information is available regarding the proportion of energy distributed to each maturing oocyte, nor regarding how variable the number and proportions of energy fluxes are within and between individuals. To complicate matters even further, follicle ovulation is controlled by pituitary gland and the follicles themselves, which are under control of the lighting schedule (Nordstrom, 1973, The VetMed webpage, 2019). Ultimately, in the expanded module, we would obtain a $t_e$ (or $\dot {k}_R$) distribution as a model output not its input, but much of the sources of that distribution would be guessed. At this point, still much information is lacking for a meaningful model extension.

### Hypothesis selection for energy allocation

Finally, one of the major study findings is that the energy ingested by the hens in the preparation for, and during, reproduction is *not* used directly and exclusively for egg production, but instead to cover the additional costs of maintenance incurred by the reproduction as well as for laying eggs, (H2 or Hypothesis 2 in this study). This conclusion could not have been attained without a mechanistic (process) model tracking simultaneously the processes of food and energy acquisition and its subsequent utilization or a very specifically designed experiment (e.g. Pick *et al.*, 2016, Ward & Bryant, 2006). One of the major reasons is that birds employ various strategies to optimize energy utilization prior to and during the reproductive season, and rarely all types of studies are available for all types of birds (Pick *et al.*, 2016, Vezina & Salvante, 2010, Ward & Bryant, 2006). Studying behaviour and tracking food and energy expenditure is not straightforward in such a diverse group as birds, so it is not surprising that answering a seemingly simple question of ‘What do the quail do with the extra food they ingest?’ posed a worthy puzzle.

The choice of H2 was based on available data and metabolic patterns that matched data reported in literature. Needless to say, a major caveat is the lack of adult weight data precisely for the egg-laying phase. Understandably, the study design is aimed at avoiding additional stress to the birds (OECD, 1984); however, this does cause gaps in data and a source of uncertainty when choosing a metabolic pathway module. Furthermore, model formulation of H2 deviates from standard DEB theory where cost of behaviour, which in large part constitutes metabolic costs of breeding (Vezina & Salvante, 2010), is accounted for via the ‘standard’ somatic maintenance $[\dot {p}_M]$ (Kooijman, 2010). The up-regulation of feeding and, consequently, extra assimilates associated with reproduction, are in H2 linked to extra-maintenance costs that occur due to reproduction. This deviation makes sense, in light of the extra metabolic costs changing the value precisely of the $[\dot {p}_M]$ parameter during the breeding period. The extra maintenance costs in this model formulation can be interpreted as the cost of tissue remodeling combined with higher-than-average maintenance cost of the reproductive machinery. Combined with ‘standard’ maintenance and overheads of growth, assimilation and reproduction, all of which are larger in H2 formulation, the total respiration is increased (Table 4). Of course, it helps that the predictions for increased respiration obtained by this formulation match the observations for a higher resting metabolic rate during reproduction reported for, e.g. Japanese quail (Pick *et al.*, 2016), great tit (Nilsson & Raberg, 2001), and European starling (Vezina & Williams, 2002), while the alternative hypothesis (H1) produced the opposite-than-observed trend (Table 4). Interestingly, the model predictions for H2 O$_2$ consumption calculated with and without feeding suggest almost three times higher (relative to no-feeding scenario) O$_2$ consumption when contribution of feeding to respiration is taken into account. The higher O$_2$ consumption (H2) is only half of that calculated based on results in Pick *et al.* (2016) (Table 4), however, if we assume a similar metabolism of the bobwhite and Japanese quail, our results suggest that the feeding restrictions in the original experiment were not long.

Both females and males change food and body composition prior to and during the reproduction season (Brennan *et al.*, 2020), and require a certain proportion of protein in their diet for optimum growth and reproduction (Aboulela *et al.*, 1992, Brennan *et al.*, 2020), but also maintenance (Brennan *et al.*, 2020). The male gonads also enlarge and then atrophy every reproductive season (Vezina & Salvante, 2010). All of this implies higher metabolic rates during reproduction (compared with non-reproductive period) also for males. We link the male cost of reproduction to the cost of the sperm ($E_{\mbox {\small {sperm}}}$), which is estimated as being substantial (10–15 kJ, Table 2). However, we do not model the metabolic changes of males in detail, mostly due to the lack of specific data on reproduction (e.g. quail sperm energy content, body mass throughout reproduction, links between sperm energy content or motility and survival of chicks), and the focus on the study being on females. Females are not only the egg-laying half of the pair, but also exhibit more pronounced effects, as the period of egg-laying seems to be especially energetically hard on females: in the post-reproductive phase females lose a larger proportion of weight than males do, which is linked to both a reduced food intake outside of egg-laying regime, but also ‘due to the regression of the female reproductive system [...] and to the additional stress of egg production [...]’ (Wilson *et al.*, 1979). Quantifying reproduction-linked metabolic changes in males might become more relevant in the future (for example, Vezina & Salvante, 2010). For birds like quail, such a study should probably explicitly include a behavioural aspect, as males seem to exhibit strong competition even in captivity (Quinn *et al.*, 2009).

**Concluding remarks**

To summarize, model predictions capture well the whole ontogeny of a generalized northern bobwhite quail, offer a clear and consistent energy pathway for extra energy assimilated during up-regulated feeding of quail, and the model is able to reproduce most of the variability observed in the available data. Reliable models with a capacity to predict physiological responses of individuals are relevant for experimental setups studying effects of various natural and anthropogenic pressures on the quail as a bird model organism, and also for wild quail management and conservation (Chown, 2012).

An important aspect of this study is that we use data on both wild (to an extent) and captive reared quail to parameterize the general life-cycle model, but focus on the latter type of data for much of the egg-laying module and inter-individual variability analysis. Consequently, our conclusions are partly based on laboratory data and, to a large extent, on data collected on reared birds. This, however, does not limit the applicability of the model to only captive reared birds, because captive reared birds share the same species-specific characteristics with their wild conspecifics. In other words, the model *parameterization* has indeed be carried out by relying heavily on laboratory data, but the model *design* is applicable to wild and captive reared quails, as well as other bird species. Namely, while parameter values are quite likely to differ between the wild and captive reared types (AmP, 2021), the rules for energy allocation are quite likely to be similar. It is likely that other closely related birds acquire, assimilate, and allocate their energy in a very similar way to quail, albeit with different parameter values. This assumption is supported by the fact that all birds in the collection (currently 503 species) as well as about 800 other species, are described well by the same type of the model (standard or ‘std’ DEB model, AmP, 2021). By specifying the egg-laying module in more detail, we hope to get a step closer to understanding the underlying mechanisms — in terms of energy and mass fluxes — in adult quail, and adults of other (closely related) birds.

The significance of having a reliable predictive model becomes evident when one needs to make predictions (Wood *et al.*, 2018), and for birds this is becoming increasingly more relevant as they are facing multiple threats due to habitat destruction, climate change and predators (Buchanan *et al.*, 2009, Doherty *et al.*, 2015, Evans, 2021, Vickery *et al.*, 2000). Use of available resources and energy depends on the size of the individuals, as does their potential to adapt to pressures (Chown, 2012). Current pressures are not only environmental degradation and pollution (Buchanan *et al.*, 2009, Reyna & Burggren, 2017, Seewagen, 2010, Vickery *et al.*, 2000), but, for game birds like quail, also hunting, which generally takes out the largest individuals of the species (Allendorf & Hard, 2009, and see Chown, 2012, review). Therefore, the knowledge of energy pathways and knowing how much energy is involved in a reproductive event — either in a rearing facility or in nature — is of utmost importance. In that context, our work is a valuable tool and step forward towards comprehensive understanding and management of the northern bobwhite quail and related species with similar characteristics. Importantly, it is also a major step towards trait-based conservation (Chown, 2012, Wood *et al.*, 2018), which makes it a most valuable tool in the emerging field of conservation physiology.
